# Thermal physiology of Amazonian lizards (Reptilia: Squamata)

**DOI:** 10.1371/journal.pone.0192834

**Published:** 2018-03-07

**Authors:** Luisa M. Diele-Viegas, Laurie J. Vitt, Barry Sinervo, Guarino R. Colli, Fernanda P. Werneck, Donald B. Miles, William E. Magnusson, Juan C. Santos, Carla M. Sette, Gabriel H. O. Caetano, Emerson Pontes, Teresa C. S. Ávila-Pires

**Affiliations:** 1 Museu Paraense Emílio Goeldi, Belém, Pará, Brazil; 2 Sam Noble Museum, University of Oklahoma, Norman, Oklahoma, United States of America; 3 University of California Santa Cruz, Santa Cruz, California, United States of America; 4 Universidade de Brasilia, Brasilia, Distrito Federal, Brazil; 5 Instituto Nacional de Pesquisas da Amazônia, Manaus, Amazonas, Brazil; 6 Ohio University, Athens, Ohio, United States of America; 7 Brigham Young University, Provo, Utah, United States of America; Clemson University, UNITED STATES

## Abstract

We summarize thermal-biology data of 69 species of Amazonian lizards, including mode of thermoregulation and field-active body temperatures (T_b_). We also provide new data on preferred temperatures (T_pref_), voluntary and thermal-tolerance ranges, and thermal-performance curves (TPC’s) for 27 species from nine sites in the Brazilian Amazonia. We tested for phylogenetic signal and pairwise correlations among thermal traits. We found that species generally categorized as thermoregulators have the highest mean values for all thermal traits, and broader ranges for T_b_, critical thermal maximum (CT_max_) and optimal (T_opt_) temperatures. Species generally categorized as thermoconformers have large ranges for T_pref_, critical thermal minimum (CT_min_), and minimum voluntary (VT_min_) temperatures for performance. Despite these differences, our results show that all thermal characteristics overlap between both groups and suggest that Amazonian lizards do not fit into discrete thermoregulatory categories. The traits are all correlated, with the exceptions of (1) T_opt_, which does not correlate with CT_max_, and (2) CT_min_, and correlates only with T_opt_. Weak phylogenetic signals for T_b_, T_pref_ and VT_min_ indicate that these characters may be shaped by local environmental conditions and influenced by phylogeny. We found that open-habitat species perform well under present environmental conditions, without experiencing detectable thermal stress from high environmental temperatures induced in lab experiments. For forest-dwelling lizards, we expect warming trends in Amazonia to induce thermal stress, as temperatures surpass the thermal tolerances for these species.

## Introduction

Body temperature (T_b_) in ectotherms influences all physiological and behavioral processes [1]. Consequently, maintenance of T_b_ within suitable limits is essential for ectotherms survival [2]. Thermoregulators actively maintain T_b_ within a restricted range of temperatures by heliothermy, i.e., by basking in the sun, or by thigmothermy, i.e., by contact with warm surfaces [3]. Thermoconformers do not actively thermoregulate, so their T_b_ parallels fluctuations in the environmental temperature [1, 4]. However, no lizard species has been shown to be a complete thermoconformer; all will move to avoid unfavorable extreme temperatures. This category is often used for species that select areas with relatively uniform temperatures, such as shaded forest, where active thermoregulation is not needed to maintain relatively stable body temperatures. Using a strictly thermoconforming strategy requires that species have broad thermal tolerances [1], and experience high variation in T_b_ throughout the day, season and geographic range.

In the field, lizards are usually active at a restricted range of T_b_. It is commonly assumed that these temperatures represent their actual thermal preferences [5]. However, laboratory experiments show that the variance in T_b_ range observed in nature for tropical lizards exceeds both the preferred T_b_ and the voluntary T_b_ range observed when the animals are subjected to thermal gradients [6–7]. Consequently, tropical lizards may already be experiencing T_b_’s at or above their physiological optima [8], putting them dangerously close to their upper thermal thresholds. These upper thermal limits are likely to be exceeded in the next few decades as a consequence of climate change [9]. An alternative interpretation is that preferred T_b_ and voluntary T_b_ reflect not only physiological limits, but are also tailored to specific activities, such as digestion, reproduction and foraging for different types of prey [1], and that laboratory studies do not fully reflect the range of motivational states. Field activity temperatures may vary seasonally, independent of variation in environmental temperatures (e.g., [10]).

The influence of ambient temperature on key physiological traits is described by thermal performance curves (TPC) [2]. A species’ thermal sensitivity can be visualized and quantified through TPCs, which reveal several important thermal properties of ectotherms. These include the optimal temperature (T_opt_), for maximal animal performance; the breadth of temperatures that results in a species performing at ≥ 80% of its optimal capacity (B_80_); and the thermal tolerance range, which is the difference between the critical thermal minimum and maximum temperatures (CT_min_ and CT_max_), i.e., the extreme temperatures that an individual can maintain locomotor function [11]. T_opt_ can vary within and among species and varies among physiological traits, according to the hypothesis of multiple physiological optima [1, 12]. Locomotor performance is one of the best-studied traits in thermal physiology, because it is related to Darwinian fitness and presumably reflects the ability to escape from predators, capture prey, and reproduce [13]. TPCs are also useful in assessing extinction risk of ectotherms. Because global warming may alter the spatial distribution of preferred microclimates [14], animals that rely on behavioral thermoregulation may experience a reduction in the time available for activity during periods when preferred microclimates become too rare to locate without overheating [15]. Restriction in activity time can result in extirpation or extinction if the remaining time is insufficient to perform all the necessary functions for successful breeding and recruitment [16].

Amazonia is a biogeographic region predicted to be strongly affected by climate change [17–18]. It covers about eight million square kilometers spread over nine South American countries [19]. Current estimates suggest that at least 210 species of lizards occur in the Amazon, although the actual diversity is poorly known [20–21]. Observed trends in the region’s climate include an overall reduction in precipitation and increased duration and intensity of droughts, especially in southern Amazonia [22], where climate change interactions with land-use change are stronger [23–24]. Recent studies indicate a long-term decreasing trend of carbon accumulation in Amazonia due to increased tree turnover and mortality rates [25]. Moreover, increased dryness may result in large-scale reductions in biomass, carbon uptake and net primary productivity [26]. Some models suggest that these changes may induce biome shifts in Amazonia, with the forest being replaced by drier vegetation associations, such as seasonal forests and savannas [27]. Therefore, recent and projected climate trends in Amazonia will likely result in a more open canopy and increased ambient temperature for forest-dwelling lizards. Despite the vastness and complexity of Amazonian habitats, thermal-physiology data for Amazonian lizards are limited, with most studies scattered among the major groups of Squamata. Most data are focused on reports of field-active T_b_ and there have been few controlled experiments on preferred or optimal T_b_.

We aim to provide the first comprehensive summary of thermal physiology characteristics of Amazonian lizards, which is essential to enhance our understanding of the effects of global warming on current and future lizard diversity in this megadiverse region. We first characterize patterns of variation in T_bs_ of Amazonian lizards (including some species that occur peripherally, at the ecotone between the Amazonian rainforest and the savanna-like Cerrado, an ecophysiological tension interface). We also provide new data on the thermal biology of some of these species and summarize the information on lizards’ modes of thermoregulation. Moreover, we next analyze evolutionary trends among thermal and physiological traits by examining the consistency of trait variation with phylogeny (i.e., phylogenetic signal) and the correlations among traits after controlling for the influence of phylogeny.

## Material and methods

### Literature review

We carried out a literature survey for data on seven physiological traits of Amazonian lizards: field-active T_b_, preferred temperature (T_pref_), minimum and maximum voluntary temperatures (VT_min_ and VT_max_), critical thermal minimum and maximum (CT_min_, CT_max_) and the optimal temperature for locomotor performance (T_opt_). Only data on T_b_ were available. Because some species have distributions extending beyond Amazonia into other biomes, our review extended beyond Amazonia, and included species from the Atlantic Rainforest, Caatinga, and Cerrado regions of Brazil, as well as the Lavrado, a savanna enclave in northeastern Roraima, Brazil. We also included data from lizards occurring in tropical forests of Central America that have similar ecophysiological traits. Species were classified as thermoregulators or thermoconformers based on whether the studies indicated they were heliotherms (thermoregulators) or non-heliotherms (defined here as thermoconformers). We also reviewed the literature to search for substrate (T_sub_) and air-temperature (T_air_) data associated with T_b_, and obtained 45 studies from the last 50 years, with reported T_b_’s from 62 species occurring in Amazonia.

### Field data

**Fig 1:**
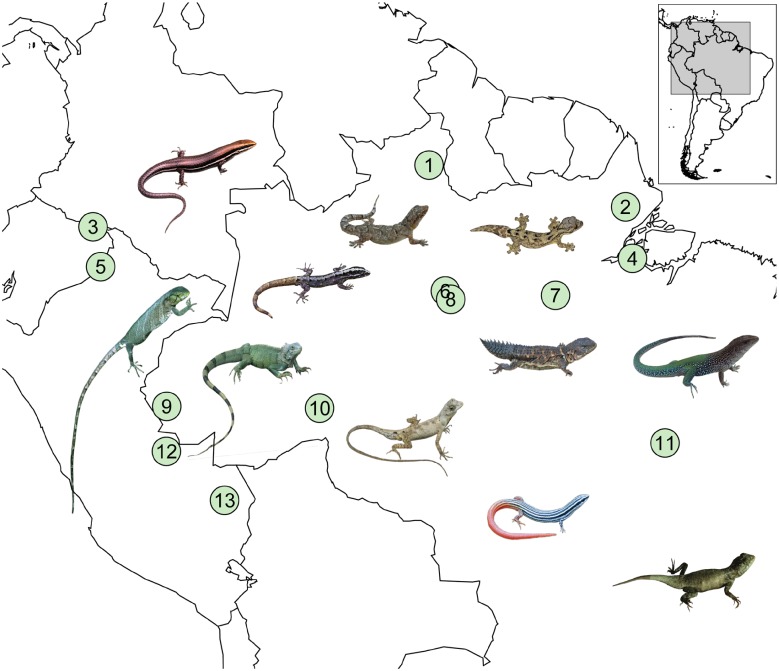
Thermal traits’ sampling localities. Body-temperature data were collected between 1993 and 1999 and thermal preference and performance data were collected between 2014 and 2016. Lizards are illustrative of the eleven families found in the field. Numbers are representative of the localities, as follows: 1) 30km NW of Caracaraí (Roraima, Brazil, 2°50’N, 60°40’W); 2) Floresta Nacional do Amapá (Amapá, Brazil, 0°55’N, 51°36’W); 3) Estación Biológica de la Pontifícia Universidad Católica (Sucumbíos Province, Quito, Ecuador, 0°0’N, 76°10’W); 4) Floresta Nacional de Caxiuanã (Pará, Brazil, 1°44’S, 51°27’W); 5) Yasuni National Park (Ecuador, 1°5’S, 75°55’W); 6) Reserva Florestal Adolpho Ducke (Manaus, Brazil, 2°57’S, 59°55’W); 7) Agropecuária Treviso (Pará, Brazil, 3° 9’ S, 54° 50’W); 8) SE of Manaus (Amazonas, Brazil, 3°20’S, 59°4’W); 9) Juruá River Basin (Acre, Brazil, 8°15’S, 72°46’W); 10) Ituxi River (Amazonas, Brazil, 8°20’S, 65°43’W); 11) Centro de Pesquisas Canguçu (Pium, Tocantins, Brazil, 9°56’S, 49°47’W); 12) Parque Estadual Guajará–Mirim (Rondônia, Brazil, 10°19’S, 72°47’W); and 13) Los Amigos Biological Station (Peru, 12°34’S, 70°6’W).

We completed our dataset with data collected by the authors throughout the year. First, we included T_b_ data on eight species collected from 1993 to 1999 in seven localities in Amazonia: Estación Biológica de la Pontifícia Universidad Católica (Quito), within the Reserva de Producción Faunística Cuyabeno (Sucumbíos Province, Ecuador, 0°0’N, 76°10’W); Juruá River Basin, ca. 5km north of Porto Walter (Acre, Brazil, 8°15’S, 72°46’W); Ituxi River (Amazonas, Brazil, 8°20’S, 65°43’W); 30km NW of Caracaraí (Roraima, Brazil, 2°50’N, 60°40’W); Parque Estadual Guajará–Mirim, on the Formoso River (Rondônia, Brazil, 10°19’S, 72°47’W); SE of Manaus, on the margin of the Amazon River (Amazonas, Brazil, 3°20’S, 59°4’W); and Agropecuária Treviso, 101 km S and 18 km E of Santarém, close to Curuá-Una River (Pará, Brazil, 3° 9’ S, 54° 50’W) (Fig 1). For the species from these localities, cloacal temperatures were measured from adult individuals with Miller & Weber quick reading cloacal thermometers (resolution of 0.2°C). Literature and empirical data provide information on the general thermal characteristics of each species, and we do not address within-species variation due to factors such as reproduction, digestion and infection.

Ecophysiological data were collected in strict accordance with the recommendations in the Guide for the Care and Use of Laboratory Animals of the National Institutes of Health. The protocol was approved by the Comissão de Ética no Uso de Animais (CEUA)–INPA (Permit Number: 029/2014), CEUA–MPEG (Permit Number: 01/2015), and CEUA UnB (33716/2016). All collecting in Brazil was done under pertinent IBAMA (073/94-DIFAS) and SISBIO (13324–1, 49241, 50381, 44832–1) permits. All efforts were made to minimize discomfort to research animals.

We collected thermal-preference and performance data on 27 species between 2014 and 2016 in six localities, five in Amazonia—Floresta Nacional do Amapá (Amapá, Brazil, 0°55’N, 51°36’W), Floresta Nacional de Caxiuanã (Pará, Brazil, 1°44’S, 51°27’W), Reserva Florestal Adolpho Ducke (Manaus, Brazil, 2°57’S, 59°55’W); Los Amigos Biological Station (Peru, 12°34’S, 70°6’W), and Yasuni National Park (Ecuador, 1°5’S, 75°55’W), and one in the Amazonia-Cerrado ecotone—Centro de Pesquisas Canguçu (Pium, Tocantins, Brazil, 9°56’S, 49°47’W) (Fig 1). Specimens were captured by active search or with pitfall traps checked twice daily. Lizards were kept in captivity for a period of no more than three days, and were released at their site of capture after a recovery time of at least four hours after the last trial. While in captivity, animals were housed individually in plastic containers with air holes and a damp cloth for moisture, without access to food. We measured T_pref_, CT_min_, CT_max_, and thermal performance curves on captive lizards. Table 1 shows the number of individuals used in each test by species. We measured snout-vent length (SVL) to 0.1mm with a Vernier caliper. A few voucher specimens of each species were euthanized with a lethal dose of Tiopental anesthetics, fixed in 10% formalin, and permanently stored in 70% ethanol. Voucher specimens were deposited in the Herpetological Collections of Museu Paraense Emílio Goeldi (MPEG), Pará, Brazil; Instituto Nacional de Pesquisas da Amazônia (INPA), Amazonas, Brazil; Universidade de Brasília, Distrito Federal (CHUNB), Brazil; Monte L. Bean Life Science Museum, Utah, USA; and Museo de Historia Natural de la Universidad Nacional Mayor de San Marcos, Lima, Peru.

**Table 1:** Thermal traits of Amazonian lizards.

Species	ActP	EAR	T_b_	T_sub_	T_air_	T_pref_	VT_min_	VT_max_	CT_min_	CT_max_	T_opt_	SVL	SR
		LAR											
GEKKONIDAE													
*Hemidactylus mabouia* * [75]	N	-	27.4 (116)	25.6	24.9	27.4 (10)	26.5 (10)	28.7 (10)	10.6 (9)	36.1 (9)	-	50.3 (10)	A, L
			20.6–29.6			18.9–33.8							
*Hemidactylus palaichthus* [76–77]	N	18:00h	26.7 (76)	25.4	25.2	-	-	-	-	-	-	48.8 (8)	A, L
		22:00h	24.2–33.2										
PHYLLODACTYLIDAE													
*Gymnodactylus amarali* [78–79]	N	-	30.2 (28)	26.8	26.5	-	-	-	-	-	-	39.5 (370)	Ce
			26.2–34.1										
*Phyllopezus pollicaris* [80–81]	C	13:00h	28.9 (10)	29.5	28.6	-	-	-	-	-	-	-	Ca, Ce
		24:00h	27.8–36.6										
*Thecadactylus rapicauda* * [75, 82–83]	N	20:00h	26.9 (80)	26.2	26	28.0 (7)	26.9 (7)	29.4 (7)	3.1 (2)	38.4 (3)	-	110.0 (7)	A
		23:00h	24.2–28.6			21.8–33.8							
SPHAERODACTYLIDAE													
Chatogekko amazonicus * [65]	D	09:00h	27.5 (36)	27.5	27.9	23.8 (117)	22.08 (117)	25.3 (117)	9.4 (37)	38.6 (32)	25.8 (24)	20.4 (112)	A
		17:30h	24.6–30.2			16.1–39.5							
*Coleodactylus septentrionalis* [65]	D	09:00h	27.4 (50)	-	27.4	-	-	-	-	-	-	26.9 (1)	A
		15:00h	-										
*Gonatodes annularis* * [20]	D	12:30h	-	-	-	31.8 (1)	31.0 (1)	32.5 (1)	16.5 (1)	-	-	33 (1)	A
		15:30h				31–32.5							
*Gonatodes concinnatus* [83]	D	09:30h	27.0 (156)	25.5	25.9	-	-	-	-	-	-	43.3 (1)	A
		15:00h	25.2–30.3										
*Gonatodes hasemani* [84]	D	07:00h	30.6 (22)	27	26.9	-	-	-	-	-	-	39.9 (1)	A
		19:00h	28.2–33.2										
*Gonatodes humeralis* *[20, 84–86]	D	07:00h	29.2 (110)	27.3	27.3	26.0 (212)	24.8 (212)	27.2 (212)	8.7 (68)	40.9 (63)	26.0 (66)	36.7 (186)	A
		19:00h	24.8–30.4			15.2–33.9							
DACTYLOIDAE													
*Dactyloa punctata* * [87]	D	10:00h	29.2 (32)	28.1	28	27.2 (4)	23.8 (4)	30.7 (4)	8.0 (2)	39.6 (2)	-	77.9 (5)	A
		17:00h	25.8–32			24.5–29.6							
*Dactyloa transversalis* * [87]	D	08:00h	29.0 (12)	26	26.3	24.1 (1)	24.0 (1)	24.3 (1)	-	-	-	76.5 (2)	A
		16:00h	25.4–29.7			23.9–24.3							
***Norops auratus*** [76]	D	09:00h	33.9 (36)	29.9	29.2	-	-	-	-	-	-	43.9 (123)	A
		17:30h	30.2–37.2										
*Norops brasiliensis* [88]	D	08:00h	30.6 (46)	30.2	31	-	-	-	-	-	-	65.3 (36)	Ce
		17:00h	26.5–34.6										
*Norops chrysolepis* *	D	09:30h	-	-	-	29.15 (26)	28.1 (26)	30.16(26)	9.4 (19)	39.7 (19)	-	46.1 (13)	A
		16:00h				27.2–33.4							
*Norops fuscoauratus* * [89]	D	08:30h	28.6 (86)	27	27	27.02 (105)	25.75 (105)	28.23 (105)	8.4 (48)	39.8 (46)	27.8 (30)	43.6 (122)	A
		17:30h	25.7–33.8			19.2–33.1							
*Norops ortonii* *	D	08:30h	30.3 (7)	28	27.5	27.8 (14)	26.9 (14)	28.8 (14)	9.7 (4)	42.3 (3)	-	44.0 (11)	A
		16:00h	27.5–31.2			22.9–33.5							
*Norops planiceps* * [90–91]	D	-	28.3 (19)	26.1	26.2	29.1 (16)	27.5 (16)	29.3 (16)	9.6 (11)	40.3 (11)	-	55.7 (13)	A
			26.3–30.8			23.1–33							
*Norops scypheus* [20, 92–93]	D	-	27.3 (36)	26.6	26.3	-	-	-	-	-	-	-	A
			24.8–28.8										
*Norops tandai* [94]	D	08:00h	27.7 (33)	27	26.9	-	-	-	-	-	-	-	Ce
		17:00h	25.2–31.2										
*Norops trachyderma* [83, 93]	D	09:00h	27.8 (31)	26.9	26.9	-	-	-	-	-	-	53.1 (1)	A
		16:00h	25.6–29.8										
HOPLOCERCIDAE													
*Enyalioides laticeps* [88]	D	09:00h	25.6 (6)	25.3	25.7	-	-	-	-	-	-	114.0 (1)	A
		15:00h	25–26.1										
IGUANIDAE													
***Iguana iguana*** [95–96]	D	-	35.3 (6)	28.5	28.5	-	-	-	-	-	-	387.5 (1)	C
			26.7–42.4										
POLYCHROTIDAE													
***Polychrus acutirostris*** [81]	D	09:00h	35.0 (8)	32.6	30.7	-	-	-	-	-	-	125.1 (1)	Ca
		15:00h	34.2–36.4										
*Polychrus marmoratus* *	D	-	29.0 (1)	26.1	26.2	-	-	-	-	-	-	127.5 (1)	A
TROPIDURIDAE													
*Plica plica* * [97–99]	D	08:00h	29.1 (56)	27.8	27.4	26.2 (23)	25.2 (23)	27.4 (23)	9.3 (17)	41.5 (17)	27.4 (10)	109.1 (21)	A
		18:00h	25.6–33.8			18.4–33							
*Plica umbra* * [83, 100]	D	09:30h	28.7 (38)	27.6	27.6	27.2 (15)	25.9 (15)	28.3 (15)	9.9 (10)	39.7 (10)	-	85.0 (19)	A
		14:00h	24.8–32.0			16.2–31.3							
*Stenocercus roseiventris* *	D	09:00h	28.2 (3)	27.6	28	-	-	-	-	-	-	85.0 (1)	A
		14:30h	26.2–32.0										
*Tropidurus hispidus* * [99, 101]	D	10:30h	34.2 (130)	33.1	30.3	29.1 (2)	28.8 (2)	30.1 (2)	13.2 (2)	43.1 (2)	-	96.8 (82)	A, Ce
		17:00h	30.6–39.6			27.8–30.3							
***Tropidurus insulanus*** [79, 97]	D	-	34.5 (51)	30	28.1	-	-	-	-	-	-	75.2 (-)	Ce
			-										
***Tropidurus oreadicus*** [102]	D	08:30h	32.9 (159)	30.4	28.7	-	-	-	-	-	-	-	Ce
		18:00h	32.0–38.1										
***Uracentron flaviceps*** [103]	D	08:30h	31.2 (22)	27.9	27.6	-	-	-	-	-	-	107.3 (11)	A
		17:30h	25–36.7										
*Uranoscodon superciliosus* *	D	11:00h	27.8 (24)	27.3	27.1	28.3 (7)	27.0 (7)	29.5 (7)	11.3 (5)	39.5 (5)	-	108.9 (7)	A
		16:00h	24.8–30.1			26.8–33.6							
SCINCIDAE													
*Copeoglossum nigropunctatum* * [100]	D	10:00h	33.2 (121)	29.9	28.7	29.1 (23)	28.0 (23)	30.3 (23)	10.4 (19)	44.3 (19)	27.3 (11)	92.5 (24)	A
		16:00h	28.0–37.4			22.2–33.5							
***Notomabuya frenata*** [98, 104]	D	07:00h	31.8 (145)	26.2	26.4	-	-	-	-	-	-	56.7 (56)	AF
		18:00h	21.7–37										
***Varzea bistriata*** [105]	D	08:00h	32.9 (11)	-	-	-	-	-	-	-	-	87.2 (24)	A
		16:00h	27.6–36.8										
GYMNOPHTHALMIDAE													
*Alopoglossus angulatus* * [106]	D	10:00h	27.3 (10)	25.1	25.6	23.8 (3)	19.9 (3)	25.6 (3)	9.0 (2)	37.2 (2)	-	49.0 (3)	A
		17:00h	25.4–33.0			20.3–27.5							
*Alopoglossus atriventris* [20,106–108]	D	09:00h	28.2 (12)	25.9	26.4	-	-	-	-	-	-	53 (1)	A
		18:00h	24.9–34.0										
Arthrosaura kockii * [20]	D	10:00h	-	-	-	26.5 (43)	25.3 (43)	27.4 (43)	10.1 (29)	43.4 (28)	25.2 (12)	30.3 (42)	A
		15:00h				20.0–30.1							
Arthrosaura reticulata* [20]	D	09:00h	27.0 (34)	25.9	26.1	24.4 (39)	23.4 (39)	25.6 (39)	8.6 (19)	36.1 (19)	25.6 (8)	50.7 (39)	A
		16:30h	23.8–28.2			15.4–27.7							
*Cercosaura argulus* * [109]	D	09:00h	29.0 (13)	27.2	27.3	25.8 (1)	25.6 (1)	25.8 (1)	-	-	-	34.5 (1)	A
		16:00h	26.2–30.8			25.3–28.0							
Cercosaura eigenmanni * [20, 91]	D	09:30h	29.7 (20)	27.7	27.4	25.3 (3)	24.9 (3)	25.4 (3)	-	-	-	45.5 (1)	A
		15:30h	27.6–31.9			25.0–25.7							
***Cercosaura manicata*** [109]	D	-	29.7 (2)	28	28	-	-	-	-	-	-	-	A
Cercosaura ocellata * [20, 91]	D	09:30h	28.1 (13)	-	-	28.4(3)	27.4 (3)	29.6 (3)	-	-	-	52.2 (2)	A
		15:00h	24–30.2			26.6–29.8							
*Cercosaura oshaughnessyi* [20, 83, 91]	D	09:00h	29.5 (13)	26.7	26.7	-	-	-	-	-	-	37.4 (1)	A
		15:30h	26.2–30.8										
Iphisa elegans* [20, 109]	D	09:00h	28.2(1)	30.2	29.5	25.4 (2)	24.6 (2)	26.1 (2)	3.1 (1)	38.4 (1)	-	46.7 (3)	A
		17:00h				21.3–29.9							
*Leposoma guianense* * [20]	D	09:00h	-	-	-	25.6 (26)	23.6 (26)	28.0 (26)	10.4 (15)	37.3 (11)	-	28.5 (25)	A
		17:00h				20.8–28.6							
*Leposoma osvaldoi* * [20]	D	09:30h	-	-	-	24.3 (11)	23.1 (11)	25.8 (11)	9.9 (9)	36.4 (8)	-	29.6 (11)	A
		15:30h				20.5–32.4							
*Leposoma percarinatum* * [20]	D	08:00h	29.7 (8)	26.7	26.6	24.1 (49)	22.3 (49)	25.8 (49)	9.0 (30)	38.7 (30)	28.8 (17)	32.3 (32)	A
		17:00h	28.2–31.8			13.4–31.9							
***Micrablepharus maximiliani*** [95, 98, 102]	D	10:00h	29.1 (4)	-	-	-	-	-	-	-	-	36 (1)	Ce
		18:00h											
*Potamites ecpleopus* [20, 110]	D	08:30h	27.0 (63)	25.5	26	-	-	-	-	-	-	61.8 (1)	A
		18:00h	23.8–31.8										
*Potamites juruazensis* [110]	D	08:30h	26.4 (8)	26.1	26	-	-	-	-	-	-	41.6 (1)	A
		16:30h	25.4–27.8										
*Tretioscincus agilis* * [20]	D	09:00h	-	-	-	27.8 (10)	26.8 (10)	29.4 (10)	9.3 (7)	40.2 (7)	-	52.2 (15)	A
		14:00h				23.5–33.0							
TEIIDAE													A
Ameiva ameiva * [111–112]	D	10:00h	37.4 (283)	32.2	30.3	29.2 (68)	27.7 (67)	30.4 (67)	11.0 (47)	46.1 (45)	34.5 (20)	127.7 (68)	A
		16:00h	26.2–41.7			18.5–38.3							
***Ameiva parecis*** [113]	D	09:00h	38.2 (54)	34.8	30.9	-	-	-	-	-	-	64.5 (1)	A
		13:00h	31.0–42.0										
*Cnemidophorus cryptus* * [113]	D	09:30h	39.4 (11)	37.4	32.3	27.6 (40)	26.7 (40)	28.4 (40)	8.4 (20)	50.1 (20)	30.5 (20)	65.4 (40)	A
		16:00h	34.6–44.4			14.5–32							
***Cnemidophorus gramivagus*** [79, 113]	D	09:00h	37.6 (42)	-	-	-	-	-	-	-	-	56 (1)	A
		14:00h	30.4–40.0										
***Cnemidophorus lemniscatus*** [76]	D	09:00h	37.6 (96)	37.2	31.6	-	-	-	-	-	-	64.2 (1)	A
		16:00h	29.1–40.7										
***Crocodilurus amazonicus*** [114]	D	11:00h	31.2 (30)	30.4	27.6	-	-	-	-	-	-	220.0 (2)	A
		16:00h	27.4–35.0										
***Dracaena guianensis*** [114]	D	-	32.2 (1)	29	29	-	-	-	-	-	-	330 (1)	A
***Kentropyx altamazonica*** [77]	D	09:30h	36.0 (66)	30.9	29.4	-	-	-	-	-	-	85 (1)	A
		15:30h	28–41.2										
Kentropyx calcarata * [47]	D	10:00h	34.7 (99)	30.6	29	34.2 (97)	32.9 (97)	35.6 (97)	11.9 (31)	41.7 (30)	-	100.8 (145)	A
		16:00h	28.7–41.0			23.3–39.1							
***Kentropyx pelviceps*** [83, 115]	D	10:00h	35.1 (143)	29.9	28.6	-	-	-	-	-	-	104.3 (32)	A
		16:00h	26–40.5										
***Kentropyx striata*** [76,116]	D	09:00h	35.7 (111)	30.3	29.5	-	-	-	-	-	-	91.6 (110)	L
		17:00h	28.8–41.0										
***Salvator merianae*** [81]	D	09:00h	35.0 (8)	32.6	31.2	-	-	-	-	-	-	-	Ca
		15:00h	34.2–36.4										
*Tupinambis longilineus* *	D	-	35.5 (3)	29.8	30	-	-	-	-	-	-	196.0 (1)	A
			33.5–37.2										
*Tupinambis quadrilineatus* *	D	-	37.2 (1)	-	-	-	-	-	-	-	-	-	A
***Tupinambis teguixin*** [76, 117]	D	09:30h	33.2 (11)	30.4	28.9	-	-	-	-	-	-	362.1 (8)	L
		15:30h	26.1–37.2										

Number of analyzed specimens in parenthesis after mean values. Species in bold are consider heliotherms in literature. Numbers in brackets are the references for the data obtained from literature, and asterisks (*) represents newly data provided in this study. ActP = Activity period (D = Diurnal, N = Nocturnal, C = Cathemeral); EAR = Earliest Activity Record; LAR = Latest Activity Record; T_b_ = body temperature (mean and range); T_sub_ = substrate temperature (mean); T_air_ = air temperature (mean); T_pref_ = preferred temperature (mean and range); VT_min_ = minimum voluntary temperature; VT_max_ = maximum voluntary temperature; CT_min_ = critical thermal minimum; CT_max_ = critical thermal maximum; T_opt_ = optimal temperature; SVL = snout-vent length; SR = study region (A = Amazonian rainforest; AF = Brazilian Atlantic Forest; C = Central America; Ca = Brazilian Caatinga; Ce = Brazilian Cerrado; L = Brazilian Lavrado).

We characterized the thermal biology of captured lizards with the following protocol. We measured the lizards’ T_b_ by using infrared thermometers, focusing the laser on the mid-portion of the animal’s ventral side, with approximately 15cm between animal and thermometer. We validated the use of body temperatures based on infrared thermometers with data on *Zootoca vivipara*, with high correlation between core and surface temperatures (0.85; n = 34, P<0.001). This species is of similar size to most of the lizards in our data set. Smaller species should present even higher correlations between core and surface temperatures, and we did not include any large species in the laboratory tests. T_pref_ and voluntary upper (VT_max_) and lower (VT_min_) temperatures were measured using a thermal gradient. Lizards were placed for 2 hours in plywood tracks 1m in length and 40cm wide, with a photothermal gradient of 15–40°C generated across each track using ice at one end and a heating lamp ~100W full spectrum at the other (*sensu* [28]). T_b_ was measured every 3–5 minutes, and T_pref_ was estimated as the mean of all T_b_ values recorded. The first measurement was made after five minutes of the animals’ positioning inside the track, to allow lizards to get acclimated to the track and reach their preference. VT_max_ and VT_min_ for each individual during this interval were estimated by the interquartile range of T_pref_ [29]. We measured T_pref_ for all individuals captured. Diurnal lizards were tested during the day, while nocturnal lizards were tested after sunset. Afterwards, lizards were arbitrarily chosen to undergo either the thermal tolerance or performance tests.

Critical temperatures were measured on 485 individuals of 26 species. An individual’s body temperature was decreased or increased in a chamber cooled by ice packs or heated by hot water until the animal lost its righting response. Each animal was tested for both CT_min_ and CT_max_, and heated/cooled to their T_pref_ immediately after the tests. We always measured CT_min_ before CT_max_, since the last may get the animals most impaired and thus needs that the animals have a longer recovery time. To calculate T_opt_, we measured locomotor-capacity experiments on 254 individuals. We stimulated each individual to run once at 2–7 randomly-assigned temperatures (15°, 20°, 25°, 30°, 35°, 40° and 43°C). Species that only occur in shady environments may suffer at extreme temperatures, and such species were run at 20°, 25°, 30° and 35°C. Lizards were allowed to recover at least four hours between trials. During the recovery period, we monitored their T_b_ and activity inside their containers at least once every hour, in order to assess their health and well-being after the stress tests. We only released the animals after we assessed that they had recovered their normal activity pattern. No animal died prior to the end of the experiments.

To measure performance, the experimenter manually stimulated lizards to run around a circular track [30]. A track with a 4m circumference was used for lizards with SVL≥50mm, and a track with 1m circumference was used for lizards SVL<50mm. Each trial ended when the lizard reached exhaustion and was unable to right itself when placed in a supine position. Animal performance was calculated as the voluntary distance traveled (number of times around the track x track circumference). T_opt_ was the body temperature that yielded the highest value of locomotor performance. We determined T_opt_ from the thermal performance curves.

### Analysis

We used the statistical software environment R 3.3.3 [31] for all calculations. Dependence between thermal physiology parameters of a priori classification of thermoregulation modes, SVL, families and species were analyzed by simple stepwise regression and one-way analysis of variance. Shapiro and Levene’s tests were used, respectively, to test assumptions of normality and homogeneity of variance for parametric variables. We used the Pearson correlation coefficient to determine the correlation between T_b_, T_sub_ and T_air_. For comparative analyses, we used the chronogram for Squamata estimated by Zheng & Wiens [32], which included all of the species for which we were able to assemble thermophysiological traits. Phylogenetic signal was calculated based on Blomberg’s K [33], which is an evolutionary model-based metric of phylogenetic-signal strength. A K-value of one indicates that the distribution of trait values follows the expectation of Brownian motion model of evolution along the tree [33]. This indicates that trait variance among species accumulates in direct proportion to their divergence time, as measured by the branch lengths separating them in a phylogenetic tree [34–35]. Values of K<1 indicate that traits are less conserved than expected, an indication of adaptive evolution, whereas values of K>1 indicate that trait values are more conserved than expected by Brownian motion evolution. We used phytools [36] to calculate Blomberg’s K and to measure the phylogenetic pairwise correlations between all thermal traits.

TPC’s were generated for each species using the packages ggplot2 [37], grid [31], mgcv [38] and proc [39] to do a Generalized Additive Mixed Modeling (GAMM) [40]. These models use additive nonparametric functions to model covariate effects while accounting for overdispersion and correlation, by adding random effects to the additive predictor [41]. Akaike’s Information Criterion (AIC) and Bayesian Information Criterion (BIC) were used to select the best correlation structure prior to estimating the TPC. AIC measures the quality of fit of the model, penalized by model complexity, and BIC additionally considers the number of observations included in the model [42]. Lizard performance at different temperatures was the response and individual was included as a random effect. The extremes of the curve were fixed at the average CT_min_ and CT_max_ values for that species. We tested several correlation structures to select the best fit including: temporal correlation structures (autoregressive process [corAR1], continuous autoregressive process [corCAR1], and autoregressive moving average process [corARMA]) and spatial residual correlation structures (Gaussian spatial correlation [corGaus], exponential spatial correlation structure [corExp], rational quadratics spatial correlation [corRatio] and spherical spatial correlation [corSpher]). We chose the correlation structure that yielded the lowest AIC and BIC values [42].

## Results

We obtained thermal data for 69 lizard species from eleven families (Table 1), including new data on field-active T_b_, T_pref_, thermal performance and tolerance from 27 species (Table 2). Among all species with physiological data, 64 are diurnal, one is cathemeral, and four nocturnal. Based on the literature, 38 species are classified as thermoconformers, while 31 are thermoregulators.

**Table 2:** Number of specimens and taxa used as source of data reported here for the first time.

	No. specimens	No. species	No. families
T_b_	80	8	5
T_pref_ / VR	1010	27	9
Physiological Performance	254	10	6
Thermal Tolerance Range (CT_min_, CT_max_)	485	26	8

T_b_ = body temperature; T_pref_ = preferred temperature; VR = voluntary range

**Fig 2:**
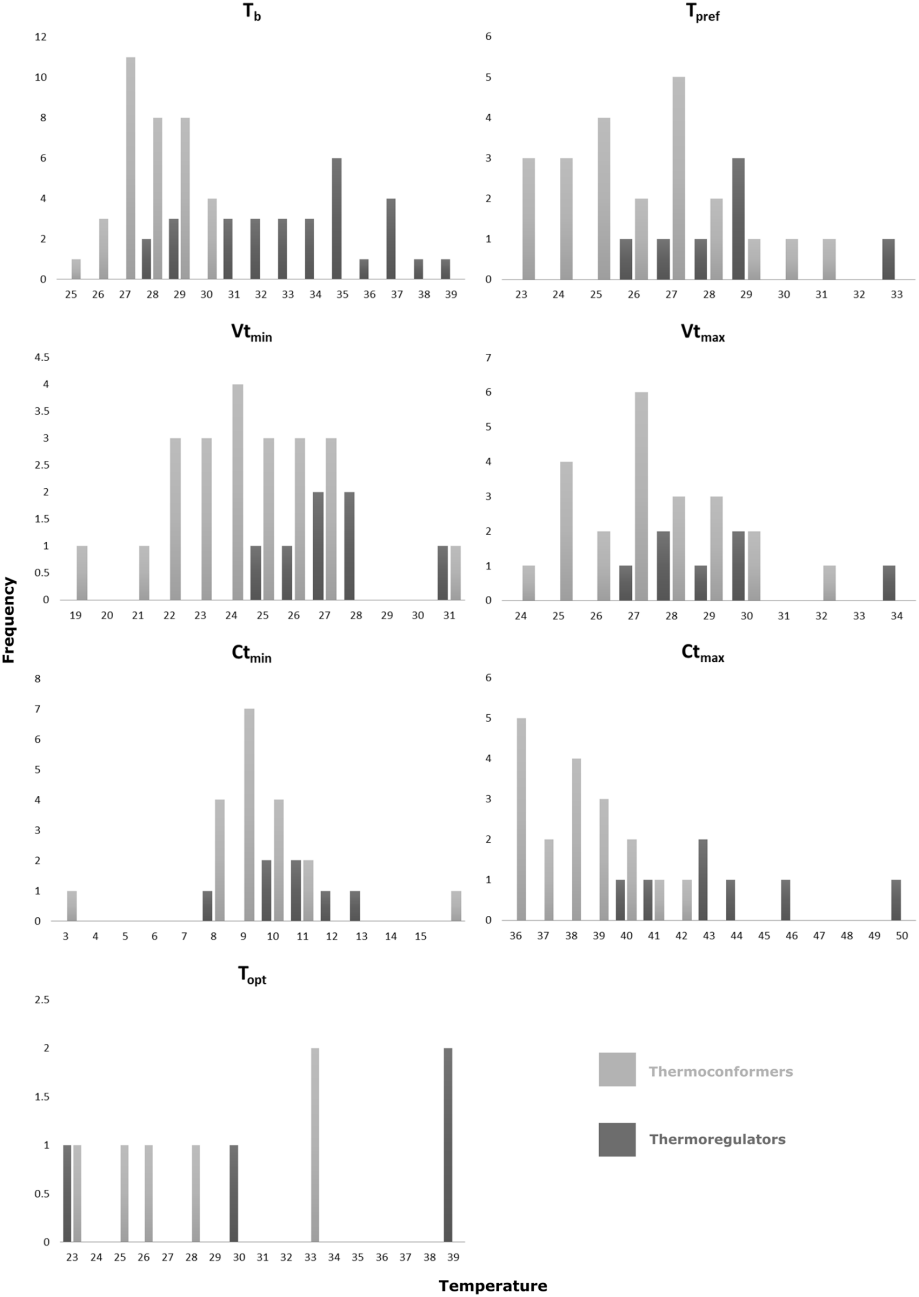
Temperature frequency distribution by thermal regulation mode for each thermal trait evaluated. Species were classified a priori as thermoregulators or thermoconformers. Values of temperature (x-axis) correspond to the mean value for each species.

**Fig 3:**
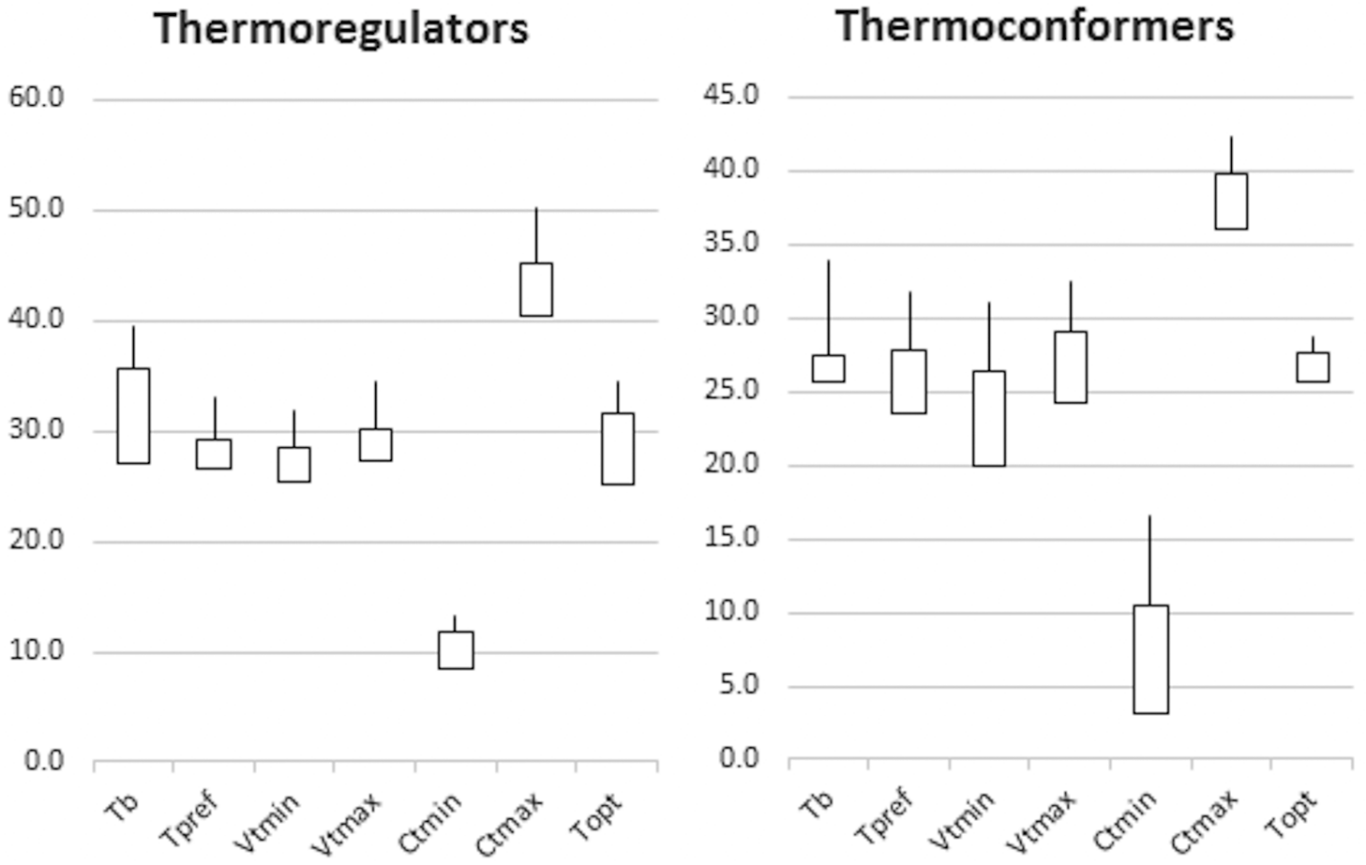
Range of evaluated thermal traits. Species were classified a priori as thermoregulator or thermoconformers. Lines indicate the maximum values.

Lizards’ body temperature was positively correlated with environmental temperature (T_b_ and T_sub:_ r = 0.80, P<0.01; T_b_ and T_air_: r = 0.67, P<0.05). Seven species generally classified as thermoconformers had T_sub_/T_air_ higher than T_b_, suggesting that these species do not gain additional heat from the environment, but may be thermoregulating by selecting lower temperatures or using evaporative cooling. A one-way ANOVA revealed significant differences in all physiological traits in relation to a priori classification of thermoregulation mode, SVL, family, and species. Fig 2 shows the range of temperatures for each evaluated trait for each thermoregulation mode. Species generally classified as thermoregulators had higher mean values for all thermal traits than those generally classified as thermoconformers, as well as larger variation in T_b_, CT_max_ and T_opt_. Variation in T_pref_, VT_min_ and CT_min_ was lower in species classified as thermoregulators and greater in species classified as thermoconformers, though mean values were relatively similar (Fig 3). In spite of these differences, our results show an overlap in most thermal traits between species classified as thermoregulators and those classified as thermoconformers, with some lizards considered thermoregulators having ranges of temperatures similar to others identified as thermoconformers. Thus, a dichotomous classification of thermoregulation mode may not be satisfactory.

We also found phylogenetic pairwise correlations between all thermal traits evaluated (T_b_, T_pref_, VT_min_, VT_max_, CT_min_, CT_max_, and T_opt_), except between (1) T_opt_ and CT_max_, and (2) CT_min_, which only correlates with T_opt_ (Table 3). Thus, selection on one thermal characteristic affects the evolution of all those considered here, except, possibly, in the two cases mentioned above. We detected significant departures from Brownian motion evolution for T_b_ (K = 0.64, P = 0.0001), T_pref_ (K = 0.49, P = 0.04), and VT_min_ (K = 0.5, P = 0.01), but not for the other thermal traits (VT_max_, K = 0.39, P = 0.21; CT_min_, K = 0.49, P = 0.12; CT_max_, K = 0.50, P = 0.079; T_opt_, K = 0.74, P = 0.17). Lizards in the family Teiidae are comprised only of species classified as thermoregulators, and had the highest values for all three thermal traits. The lowest T_b_ was found in one species of Hoplocercidae (forest-dwelling lizards), and the lowest VT_min_ was observed in Gekkonidae, both families containing only species classified as thermoconformers. Gekkonidae and Dactyloidae presented the lowest T_pref_. Although Dactyloidae is a mixed family, the only dactyloid species classified as thermoregulator in this study has no T_pref_ data available. Thus, all T_pref_ measurements for this family are from species classified as thermoconformers (six out of ten analyzed species), which may explain the low value found. Families with both thermoregulation modes tended to have intermediate mean values for all thermal parameters (Table 4). Tropiduridae had five species classified as thermoregulators with mean T_b_ 32.2°C. Of those, data on T_pref_ and VT_min_ value (29.1° and 28.8°C, respectively) were available only for *Tropidurus hispidus*. Among the three species of tropidurids classified as thermoconformers, the mean values of T_b_, T_pref_ and VT_min_ were 28.5°, 27.5° and 26.3°C, respectively. Four gymnophthalmids were classified as thermoregulators and they had mean values of T_b_, T_pref_ and VT_min_ of 29.4°, 27.5° and 25.8°C, respectively, while the other 13 species that are considered thermoconformers had mean values of T_b_, T_pref_ and VT_min_ of 28.2°, 25.1° and 23.8°C, respectively. These results reflect the among-family pattern where families with species considered to be thermoregulatory had higher mean values of T_b_, T_pref_ and VT_min_ than families which only have species classified as thermoconformers.

**Table 3:** Correlation between thermal traits (r, P).

	CT_min_	T_b_	T_opt_	T_pref_	VT_max_	VT_min_
CT_max_	0.19, 0.65	0.84, 0	0.25, 0.241	0.57, 0.002	0.57, 0.002	0.57, 0.002
CT_min_	-	0.19, 0.21	0.78, 0.005	0.19, 0.72	-0.14, 0.49	-0.20, 0.84
T_b_	-	-	0.62, 0.05	0.60, 0.002	0.59, 0.002	060, 0.0014
T_opt_	-	-	-	0.68, 0.018	0.68, 0.02	0.58, 0.05
T_pref_	-	-	-	-	0.87, 0	0.77, 0
VT_max_	-	-	-	-	-	0.85, 0

As hypotheses were independent, no correction was made for multiple tests.

**Table 4:** Mean values of body temperature (T_b_), preferred temperature (T_pref_) and minimum voluntary temperature (VT_min_) by family.

Family	T_b_	T_pref_	VT_min_	TrM
Hoplocercidae (1)	25.6	-	-	TC
Gekkonidae (2)	27	27.4	23.7	TC
Sphaerodactylidae (6)	28.3	27.8	25.8	TC
Phyllodactylidae (3)	28.7	28	27.9	TC
Gymnophthalmidae (17)	28.4	25.6	24.2	M
Dactyloidae (11)	29.2	27.4	25	M
Tropiduridae (8)	30.8	27.9	26.9	M
Polychrotidae (2)	32	-	-	TR
Scincidae (3)	32.6	29.4	28.2	TR
Iguanidae (1)	35.3	-	-	TR
Teiidae (15)	35.7	31.1	29.8	TR

The number of species we obtained data for each family is in parentheses. TrM = a priori classification of thermoregulation mode (TR = thermoregulator; M = mixed; TC = thermoconformer).

**Fig 4:**
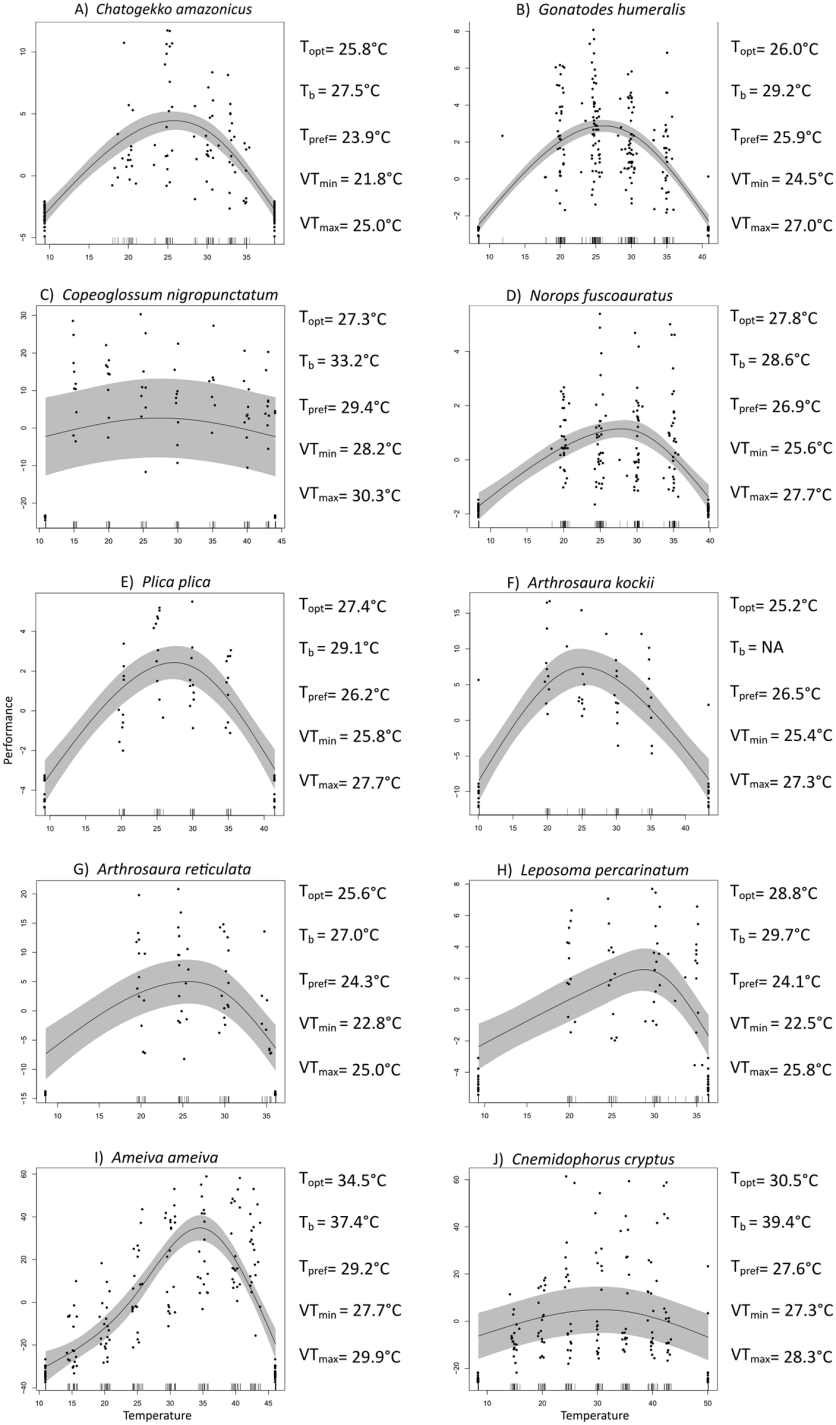
Thermal-performance curves and mean values of thermal traits. A) *Chatogekko amazonicus*; B) *Gonatodes humeralis*; C) *Copeoglossum nigropunctatum; D) Norops fuscoauratus*; E) *Plica plica*; F) *Arthrosaura kockii*; G) *Arthrosaura reticulata*; H) *Leposoma percarinatum*; I) *Ameiva ameiva*; and J) *Cnemidophorus cryptus*. Gray shaded region shows the 95% confidence interval. Black points represent the results of individual tests at different body temperatures: 15°, 20°, 25°, 30°, 35°, 40° and 43° for species classified as thermoregulators and 20°C, 25°C, 30°C and 35°C for shade-associated species classified as thermoconformers. Short vertical black lines indicate the number of trials at each temperature. Black lines at the curves’ extremes are the critical thermal minimum (CT_min_) and critical thermal maximum (CT_max_). T_opt_ = optimal temperature; T_b_ = body temperature; T_pref_ = preferred temperature; VT_min_ = minimum voluntary temperature; VT_max_ = maximum voluntary temperature.

We measured the thermal dependence of locomotor performance for ten species of Amazonian lizards (Fig 4). The best correlation structure and estimated TPC parameters for each of these ten species are presented in Table 5. The CT_max_ among all species classified as thermoregulators varied between 45–50°C. We observed small variation in the shape of the TPCs, with exception of *Copeoglossum nigropunctautm* and *Cnemidophorus cryptus*, which had almost linear curves and a high confidence interval. *Gonatodes humeralis* and *Norops fuscoauratus* are forest shade species that were classified a priori as thermoconformers whose TPC’s have broad plateaus, with CT_max_ reaching 40°C. In *G*. *humeralis*, T_opt_ is closer to T_pref_, while in *N*. *fuscoauratus* T_opt_ is closer to VT_max_. The TPC for *Arthrosaura kockii* had a plateau, which was slightly inclined at lower temperatures. *Plica plica* had a pattern similar to that of *A*. *kockii*, even though the former is considered a thermoconformer and the latter a thermoregulator. *Plica plica* had a T_opt_ closer to VT_max_, while in *A*. *kockii* T_opt_ was closer to VT_min_. For *Ameiva ameiva* there was a steep performance increase with higher body temperature, with a T_*opt*_ greater than the mean values for VT and T_pref_, and closer to T_b_. In *Cnemidophorus cryptus*, T_opt_ was greater than VT’s and T_pref_, which is similar to *A*. *ameiva*, but below its T_b_. *Chatogekko amazonicus*, *Arthrosaura reticulata*, and *Leposoma percarinatum* are found in the leaf litter and all three species have CT_max_ values near 35°C. T_opt_ for *Chatogekko amazonicus* and *A*. *reticulata’s* was close to their VT_max_, while for *L*. *percarinatum’*s T_opt_ is nearer its T_b_. T_opt_ values for both species classified as thermoregulators and thermoconformers partially overlap, similar to values for the other thermal physiological parameters analyzed.

**Table 5:** Selected correlation structures (CS) used in GAMM fitting of thermal performance curves (TPC) of Amazonian lizards.

Species	CS	BIC (R^2^)	B_80_ (°C)	T_opt_ (°C)	BP (m)	CT_min_ (°C)	CT_max_ (°C)
SPHAERODACTYLIDAE							
*Chatogekko amazonicus*	CorGaus	0.65	24.9–33.5	25.8	7.6	11.6	43.8
*Gonatodes humeralis*	CorAr1/CorARMA	0.72	11.9–35.2	26.0	5.6	8.4	41.0
SCINCIDAE							
***Copeoglossum nigropunctatum***	**CorARMA**	**0.1**	**15.0–43.0**	**27.3**	**26.2**	**9.3**	**49.3**
DACTYLOIDAE							
*Norops fuscoauratus*	CorAr1	0.43	20.1–35.4	27.8	2.98	8.5	44.2
TROPIDURIDAE							
*Plica plica*	CorSpher	0.73	25.0–35.3	27.4	6.5	7.8	44.7
GYMNOPHTHALMIDAE							
***Arthrosaura kockii***	**CorRatio**	**0.65**	**10.1–30.1**	**25.2**	**17.6**	**9.0**	**49.6**
*Arthrosaura reticulata*	CorAr1	0.48	19.6–35.1	25.6	19.1	8.6	42.3
*Leposoma percarinatum*	CorARMA	0.45	19.8–35.0	28.8	7.2	8.9	36.5
TEIIDAE							
***Ameiva ameiva***	**CorRatio**	**0.60**	**24.5–42.9**	**34.5**	**67.5**	**9.9**	**46.1**
***Cnemidophorus cryptus***	**CorARMA**	**0.17**	**14.8–43**	**30.5**	**29.3**	**10.6**	**49.1**

Values indicate Bayesian Information Criterion (BIC) and performance (range of temperatures that are ≥ 80% of optimal capacity = B_80_; optimal temperature = T_opt_; best performance = BP; critical thermal minimum = CT_min_; and critical thermal maximum = CT_max_). Thermoregulatory species are shown in bold.

## Discussion

We observed a non-significant phylogenetic correlation between T_opt_ and CT_max_, and considering that T_opt_ is correlated with all other thermal traits, this is in agreement with the argument that tolerance limits have less relevance to thermoregulation than T_opt_ [43–44]. On the other hand, this is incongruent with the results of Huey and Kingsolver [12] and Huey *et al*. [45], who found that T_opt_ and CT_max_ are correlated and tend to coevolve. Possibly this is because these studies included many shade-loving species in which the highest obtainable temperatures are well below those likely to cause physiological stress. Conversely, the correlation between CT_min_ and T_opt_ suggests that directional selection on CT_min_ will have a direct effect on locomotor performance, raising or lowering T_opt_ and the mid-level performance temperature range. This is also in accordance with the hypothesis that species that restrict their activities to deeply-shaded areas may be more limited by low rather than high temperatures [46–47].

Blomberg *et al*. [33] found relatively low values of K indicative of low phylogenetic signal for physiological traits in Australian skinks (e.g., T_pref_ and T_opt_), as we report here for T_b_, T_pref_ and VT_min_. Such a result was expected considering the importance of environment on the thermal characters of ectotherms, which in turn influences nearly all their physiological processes. These traits are apparently influenced by factors other than phylogeny, such as phenotypic plasticity, which is the ability of an organism to express different phenotypes depending on its biotic and abiotic environments [48]. This factor influences not only the thermal physiology of lizards, but also their life histories [49–51]. The low signal could also be due to local adaptation, since it indicates that these traits have not been conserved evolutionarily [52].

For the thermal parameters VT_max_, CT_min_, CT_max_ and T_opt_, we found no departure from Brownian motion evolution. Nevertheless, some of these traits may be limited by physiological constraints common to all lizards, hindering the chances of evolutionary changes that would be reflected in the phylogeny. According to Huey & Kingslover [12], if the population lacks the underlying genetic variation to shift its thermal sensitivity adequately in response to selection, we might expect that this population will not be able to adapt and evolve rapidly enough to track changes in environmental temperatures, such as those caused by the climate warming. For example, broad variation in T_pref_ can be too low [53] or additive genetic variation can be overwhelmed by maternal effects [54] to allow rapid adaptation to climate warming measured on decadal time scales [16]. Alternatively, our sampling was insufficient to detect local adaptation since we did not designed our study for that. The ages of these species are probably all in the range of millions of years, indicating that all have passed through many climatic fluctuations that affected all parts of Amazonia to some extent (e.g., [55–58]), which may have led to genetic variation within and between populations.

Thermal conditions within lowland tropical forest are likely insufficient to permit T_b_s below the level of thermoregulation found [6]. The ability to attain T_pref_ by basking in rainforest microenvironments may be limited due to lower heterogeneity in the thermal environment. All species with performance and T_b_ data available had T_opt_ < T_b_, although their T_b_’s are within the broad plateau of their TPC. This is in disagreement with Bennett [59], who suggested that T_opt_ is always higher than T_pref_ and T_b_. T_opt_ in all species is between 7.7–24.4°C below CT_max_. By comparing T_b_ with CT_max_ in 19 species with available data, CT_max_ was between 6–12°C above the mean values of T_b_, consistent with Ji *et al*.*’s* [60] study on *Sphenomorphus indicus* and Du *et al*.’s study [7] on *Eumeces elegans* males. When we consider the highest values of T_b_ measured, the difference drops to 4–11°C for most species, except *Kentropyx calcarata* and *Tropidurus hispidus*, where the difference is ~0.5°C and 3.5°C, respectively. Overall, our results partially support Hoffman and Sgró’s claim [9] that tropical lizards have their thermal traits close to their upper thermal thresholds, which are likely to be exceeded in the next few decades. Nevertheless, we measured Topt only for running speed. It may be that T_opt_ for other activities is closer to that observed for T_b_. Within the forest, it is less likely that availability of temperatures lower than T_opt_ will limit any of the species in the near future, especially in the case of heliotherms, which are probably presently more limited by low than high temperatures.

Although we found statistical differences between the thermoregulatory modes generally attributed to the species for all physiological traits, most species appear to show substantial overlap in their thermal physiology regardless of their a priori classification into thermoregulatory modes. Thus, the tropical lizard species here studied do not form discrete categories, as suggested by Pough and Gans [3]. We tested the a priori categories for thermoregulatory mode because these continue to be used to describe lizard thermoregulation, especially in relation to the predicted effects of climate change. However, the relationship between body and environmental temperatures in lizards shows a cline rather than discrete categories [3, 61] and future studies would gain by abandoning them and using continuous variables when more detailed information is available [62].

Geographic variation in climate can lead to differences in thermal physiology among species [63], so the physiological data obtained for some species outside Amazonia must be seen as an approximation of the thermal traits for those species in this region. Among all species included in this study, smaller animals had the lowest temperatures for all traits, probably due to their relatively low thermal inertia [64–65]. *Chatogekko amazonicus*, *A*. *ameiva* and *C*. *cryptus* had T_opt_ close to their T_b_’s, so these species can achieve their highest locomotor performance under current environmental conditions. In contrast, *P*. *plica* and *A*. *reticulata* achieve their T_opt_ closer to T_pref_, and *G*. *humeralis*, *C*. *nigropunctatum* and *A*. *kockii* achieve T_opt_ closer to VT_max_; in both cases, T_opt_ was lower than T_b_ obtained from field measurements. *Norops fuscoauratus* and *Leposoma percarinatum* had T_opt_ considerably above T_b_, T_pref_, and VT_max_, closer to CT_max_. For these species, T_opt_ might reflect the thermal optimum of other physiological processes, or an intermediate thermal optimum for different processes [64]. Among the species with T_b_>T_opt_, the high T_b_ likely reflects the thermal optima for other physiological process, such as digestion [11, 66–68].

We hypothesize that many tropical rainforest lizards may be affected by high environmental temperatures, considering that their locomotor performance is better at lower temperatures than they are already experiencing in field. Our inferences are in agreement with other studies with fewer species and at higher latitudes, which predict that many tropical lizards are at an imminent risk of extinction due to human induced global warming [8, 16, 69]. Additional factors are affecting large parts of Amazonia such as forest loss, degradation, and fragmentation [70]. These factors can cause rapid microclimate changes towards hotter and drier conditions that climate models are unable to predict with accuracy [71]. Most of the species in this study had some margin for an increase in average T_b_ with low cost to performance, since their T_b_s are still within the broad plateau of their TPC. However, selection is weaker on species with broad TPCs than narrow TPCs, which have a lower capacity to evolve to track changes in climate [12].

Sinervo *et al*. [16] provided the first model of the potential effects of a warming climate on species distributions based on thermal physiology. Integrative models such as those that incorporate phenotypic plasticity and genetic variability will allow projections of adaptive radiation occurring under warmer environments [72–73]. Both phenotypic plasticity and genetic variability are directly dependent on environmental conditions that allow lizards to gain and lose heat [16, 60, 74]. We expect that, with more temperature data on a higher diversity of Amazonian lizards’ species, we can better understand the effects of climate change on these animals. Also, investigations taking into account the phylogeographic history of Amazonian lizard species, many of which are known to exhibit cryptic diversity and high population structure, will be important to refine and help detect geographic divergence of thermal traits and extinction risks.

## Conclusions

This study represents the first effort to compile and provide novel thermal-biology data obtained across wide geographic ranges and taxonomic diversity of Amazonian lizards. We integrated field and literature data with phylogenetic inferences to better understand how updated ecophysiological traits can serve as a baseline to inform predictions of global warming effects on the future of rainforest lizards.

Although lizards generally classified as thermoregulators and thermoconformers show significant differences in their thermophysiological characters, our results indicate that these groups do not form discrete categories, since most species are intercalated in their thermal physiology regardless of their thermoregulation modes. Most species considered to be thermoconformers in Amazonia prefer warmer microhabitats to gain additional heat from the environment and thus cannot be considered thermoconformers in the strict sense, corroborating the idea that thermoconformity is one extreme of a continuum with thermoregulation in the other extreme [3].

Our results suggest that selection on CT_min_ will affect locomotor performance directly by amplifying or reducing T_opt_ and the range of temperatures of mid-level performance. We found no phylogenetic signal for CT_max_, CT_min_, T_opt_ and VT_max_. In turn, T_b_, T_pref_ and VT_min_ are less conserved than expected, so they appear to be influenced by factors other than phylogeny, such as strong selection or phenotypic plasticity.

Lizards are excellent models for investigating the biological effects of climate change. Although Amazonian lizards have an apparent margin for an increase in T_b_ with low cost to performance, suggesting they may show some resilience to warming, their broad TPC’s may not allow rapid evolutionary response to a quickly warming climate. More studies on the thermal physiology of Amazonian lizards are needed to obtain data representative of the high species diversity in the region so we can better understand the effects of climate change on their distribution and densities.

## Supporting information

S1 File Preferred, voluntary and critical temperatures from all individuals tested in ecophysiological experiments. T_pref_ = preferred temperature; VT_min_ = Minimum voluntary temperature; VT_max_ = Maximum voluntary temperature; CT_min_ = Minimum critical temperature; CT_max_ = Maximum critical temperature. Available on doi: 10.6084/m9.figshare.5293756.
(XLSX)
